# Positive face BOLD response and task-dependent ventral striatal functional connectivity during Go/No-go task among abstinent cannabis-using adolescents and young adults

**DOI:** 10.3389/fradm.2026.1737529

**Published:** 2026-02-01

**Authors:** Ryan M. Sullivan, Alexander L. Wallace, Carissa W. Tomas, Hailey G. Wirtz, Christine L. Larson, Krista M. Lisdahl

**Affiliations:** 1 Department of Psychiatry, University of California, San Diego, La Jolla, CA, United States,; 2 Department of Epidemiology & Social Sciences, Medical College of Wisconsin, Wauwatosa, WI, United States,; 3 Department of Psychological & Brain Science, University of Wisconsin-Milwaukee, Milwaukee, WI, United States

**Keywords:** adolescent, affective processing, cannabis, fMRI, neuroimaging, reward, ventral striatum, young adult

## Abstract

**Introduction:**

Regular cannabis use is associated with attenuated neural reward signaling, primarily measured through monetary or drug-cue tasks. Yet, minimal research has studied functional positive face processing in inhibitory control contexts, particularly amongst cannabis-using adolescent and young adults. The present study seeks to investigate functional response differences in whole-brain and ventral striatal activation, and ventral striatal functional context-dependent connectivity during positive (i.e., happy) face conditions during an emotional Go/No-go task in abstinent regular cannabis-using adolescent and young adults compared to controls.

**Methods:**

Participants (age 16–26; cannabis-using=35; control=33) underwent at least two-weeks of monitored abstinence before completing an emotional Go/No-go fMRI task. Whole-brain analyses examined blood-oxygen-level-dependent (BOLD) differences for positive (minus neutral) face conditions between groups. Bilateral ventral striatal activity was investigated in region-of-interest and task-dependent functional connectivity analysis.

**Results:**

Cannabis-using participants displayed increased left middle cingulum and decreased left supplemental motor area BOLD response during positive Go conditions. Decreased BOLD response was seen in left superior frontal region during positive No-go for cannabis-using participants. Ventral striatum activity was increased during Go and decreased during No-go conditions for cannabis-using group, with null connectivity findings.

**Discussion:**

Clusters of aberrant functional response within cannabis-using adolescents and young adults aligns with previous, but sparse, literature on positive face engagement and inhibition. Here, we demonstrate variable ventral striatum activity consistent with reward-eliciting BOLD investigations—representing importance of reward-related affective investigations—yet no connectivity differences in this sample. These findings may represent a risk for or consequence of cannabis use, as differences are still notable after two-weeks of abstinence.

## 1 Introduction

Cannabis is one of the most commonly used substances in the United States with approximately 24.6% of adolescents (grades 8, 10, 12) and 42.0% of young adults (aged 19–28) reporting past year use (1, 2). Due to increased prevalence rates in this age range, the scientific community is increasingly interested in understanding the impact of repeated and regular cannabis use on adolescent and young adult neurodevelopment.

Exogenous cannabinoids primarily consist of cannabidiol and delta-9-tetrahydrocannabinol (THC). THC use can impact the brain by interacting with the endogenous cannabinoid system (3), which includes cannabinoid receptor 1 (CB1) (4) and is primarily distributed throughout the central nervous system (5). Receptors for this system are principally involved in neuromodulation (6) and are at peak density throughout adolescence, particularly in prefrontal and limbic regions (7–9). Repeated and regular use of THC can affect CB1 binding (10) and in turn, affect downstream functional and structural changes to the brain (11). To that end, frequency of cannabis use has been related to significant reductions in the density of CB1 receptors throughout the cortex, with recovery reportedly occurring after one month of abstinence (12). Moreover, chronic exposure to cannabis has been related to alterations in cognitive control, inhibitory control, affective processing, and reward functioning (i.e., continuing beyond approximately 1-month of abstinence) (11, 13–15). Albeit the link between chronic cannabis use and affective reward processing following sustained abstinence in adolescents and young adult has yet to be fully characterized. Measuring these affective properties after abstinence is important to distinguish from short-term, relatively acute effects of cannabis, vs. longer-lasting impacts that may continue to disrupt treatment outcomes or results in impairments even after abstinence is achieved. Furthermore, this may reveal specific neuronal networks that are sensitive to longer-term impacts of chronic cannabis exposure during the adolescent and young adult years.

Among typically developing adolescents and young adults, reward circuitry centers are broadly characterized by strong signaling as they attune to evaluating and appraising rewarding stimuli (16, 17). Important regions for higher-order processes of inhibitory control (i.e., one’s prepotent ability to withhold immediate responding) (18)—including the prefrontal cortex—remain structurally and functionally underdeveloped relative to subcortical areas (19, 20). During this dyssynchronous development, it is suggested that reward signaling may be less downregulated by cortical control systems resulting in increased impulsive or reward-driven outward behavior during adolescence (21, 22). Somerville et al. (23) examined this phenomenon in adolescents and young adults with an emotional Go/No-go paradigm examining positive (i.e., “happy”) faces, where teens (aged 13–17) displayed stronger activation in the ventral striatum—a subcortical region characterized by its role in the dopaminergic system related to reward processing—compared to children and adults (23). Throughout adolescence, the ventral striatum—along with other reward-related regions—experiences an uptick in dopaminergic receptors (24, 25) and are recruited differentially depending on the context of reward processing. The striatum has been dually associated with an increase in functional activation when responding to a reward (26, 27) and a decrease in functional activation when anticipating a reward (26, 28, 29). With the onset of substance use generally occurring during adolescence (1), elucidating differences in reward processing and inhibition as it pertains to substance use risk and consequences has garnered greater attention in the broader field of addiction neuroscience (30).

It has been demonstrated both across the animal and human literature that introduction of substance use alters and reprograms key reward appraisal regions by devaluing standard rewards and thus, rewards of more salient value are required to recruit reward functioning that mirrors similar levels of activation compared to non-using counterparts (31–34). In preclinical studies, THC has been shown to decrease sensitivity to rewards and result in aberrant functioning of the reward systems (i.e., the mesolimbic dopamine system, including the ventral striatum) (35–39). Within humans, functional connectivity at rest *between* these reward regions is disrupted in cannabis-using adolescents and young adults, with non-dependent individuals demonstrating increased connectivity in prefrontal regions and dependent individuals exhibiting more subcortical region connectivity (15, 40, 41), with broad functional connectivity differences that are partially recovered after one month of abstinence in young adult cannabis-using males (42). Yet, less is known on whether functional differences—while task-engaged—may persist following abstinence and could theoretically interfere with treatment outcomes and interpersonal functioning.

Functional responding in these reward regions are typically assessed through the Monetary Incentive Delay (MID) task (26, 27, 43), with pronounced striatal hypoactivation seen when anticipating reward (44–46) and striatal hyperactivation during reward appraisal (45, 46) [see, Luijten et al. (30) for review]. This research indicates that cannabis use interplays with reward systems through exaggerated responding when engaged in reward processing, however, these findings are largely exclusive to the MID task and there is a paucity of research examining differences in functional reward responding to other pleasurable cues outside of monetary appraisal—such as through an *emotional faces* go/no-go task (23). The latter may be more ecologically valid in the context of social reward processing and interpersonal relationship functioning, concepts important for both healthy adolescent/young adult development and treatment outcomes (47, 48). Yet, minimal studies have investigated emotional go/no-go in the context of adolescent substance use, despite the focus in other prevalent psychopathologies in adolescence (e.g., depression, anxiety) (49–51). Moreover, considering the mentioned context-dependent functional connectivity differences in cannabis-using samples on fMRI task data (40, 41), continued investigation of functional co-activation while engaged in eliciting paradigms is needed. Thus, examination of reward responding activation and subsequent co-activation with reward-related areas—identified in control samples (i.e., ventral striatum) (23)—may elucidate potential risks for or effects of cannabis use on brain function.

Within investigations of cannabis use and emotional faces paradigms, studies have typically focused on negative affective stimuli processing, with cannabis-using groups showing amygdala reactivity and aberrant brain activation particularly in frontal and cingulate regions (52–55). Comparatively, only a few small-sample studies have examined functional neuroimaging differences between cannabis-using and non-using when viewing *positive affective* stimuli. Gruber et al. (52) examined functional brain response to positive face viewing in a sample of cannabis-using adults, where more activation was observed in cingulate region and less activation was observed in the left temporal lobe and amygdala when compared to non-using adults. Conversely in other studies, cannabis-using adults displayed no differences in brain activation when viewing positive situations compared to non-using adults (56); and, no associations were observed between functional brain activation to positive faces and cannabis use disorder scores in adolescents, albeit, this treatment-seeking sample comprised of increased psychopathological comorbidity prevalence (57). As these studies comprise of passive stimuli viewing, no studies have examined positive face processing while also engaging in inhibitory control contexts (i.e., Go/No-go), despite broad aberrant inhibitory findings (13, 55, 58–61). Investigations on the interplay between positive face processing and inhibitory control may shed light on problematic substance use development into adulthood, as this interplay is a key aspect of withdrawal stages within working models of addiction (33, 47). In addition, given the neurodevelopmental timing of our sample (i.e., adolescents to young adults) (23), investigating positive face processing—in the context of abstinent cannabis use—has important implications for potential downstream effects on reward-related regions (25) which have varying functional activation dependent on context of rewarding stimuli (26).

Therefore, the aims of the study were to: (1) Examine differences in whole-brain blood oxygenation level dependent (BOLD) imaging to positive faces between abstinent cannabis-using and non-using adolescents and young adults during an emotional go/no-go paradigm in both inhibitory and approach contexts (13, 23). (2) Conduct context-dependent functional analyses to examine ventral striatum activation and connectivity differences between cannabis-using and non-using groups. Whole-brain approaches were employed due to uncertainty in which specific regions may differ between groups during affective reward processing and inhibitory control while processing positive affect; we hypothesize that cannabis-using groups would demonstrate increased BOLD response in ventral striatal regions during passive viewing (i.e., Happy Go) and inhibitory control (Happy No-Go) conditions, due to aforementioned literature on reward in cannabis use. These aims were investigated in a group of nontreatment-seeking cannabis-using adolescent and young adults with no psychopathological comorbidities who underwent at least two-weeks of monitored abstinence, to mitigate effects of acute cannabis ingestion, leading up to functional imaging acquisition.

## 2 Materials and methods

### 2.1 Participants

Participants included in the present study were from a parent study (R01DA030354; PI: Lisdahl; data collection period: 9/14/2011 to 4/25/2018) examining health and neurocognitive factors among adolescent and young adult cannabis-using and non-using groups (13). Inclusion criteria consisted of right handedness, English speaking, and willingness to abstain from all substance use (except for nicotine) over a three-week period. Exclusion criteria for all participants included having an independent DSM-IV-TR Axis I disorder, major medical or neurological disorder (including metabolic disorders), current use of psychoactive medication, loss of consciousness >2 min, history of intellectual or learning disability, prenatal medical issues or premature birth (i.e., gestation less than 35 weeks), reported significant prenatal substance exposure [i.e., alcohol exposure (≥4 drinks in a day or ≥6 drinks in a week), nicotine exposure (average >5 cigarettes per day for >1 month), or any other illicit substance exposure], magnetic resonance imaging (MRI) contraindications, elevated Physical Activity Readiness Questionnaire (62)—indicating difficulty completing aerobic fitness testing (aim of the parent study), or any other excessive illicit drug use (>20 lifetime use for each drug category).

In the present analysis, cannabis-using group is categorized as those who used cannabis at least 40 times in the last year (i.e., near weekly, operationalized as a “regular-using” group) and at least 100 lifetime uses (13, 63). Non-using controls in the present analysis had no cannabis within the past year and used less than 20 times in their lifetime (63). In addition, participants must have usable task fMRI data to be included in the present analysis (*N* = 3 removed for missingness). Thus, 68 participants were included in the present study (See Table 1). The whole sample was between the ages of 16 and 26 years (M = 21.3, SD = 2.4), were equally balanced for gender (50% female), were largely non-Hispanic (80.9%), and racial identities consisted of predominantly: White (64.7%), Asian (11.8%), Multi-racial (11.8%), and Black (5.9%).

### 2.2 Procedures

All aspects of the protocol were approved by institutional IRB (#PRO00016025). Interested individuals provided written assent (minors) or consent (youth aged 18 + and parents/guardians) and were first screened for basic sociodemographic, medical, and substance use information to determine initial eligibility. The study included five in-person study sessions over the course of three weeks. The first three sessions occurred one week apart and consisted of brief neuropsychological battery [described further in Wallace, Wade, et al. (64)], behavioral measures, urinary and sweat drug toxicology testing (63). After a week following session three, sessions four and five occurred within 24–48 h of one another and consisted of conducting aerobic fitness measures, full neuropsychology battery, drug toxicology, psychological questionnaires, and brain MRI.

Throughout the study period, participants were asked to remain abstinent from cannabis, alcohol, and other substances (other than tobacco), which was confirmed through breath, urine, and sweat toxicology screening. Participants were not allowed to complete sessions four and five (i.e., MRI scan) if positive for any illicit drug use, a rise in THCOOH levels, or had a breath alcohol concentration greater than 0.000 (63). Participants who used tobacco were asked to abstain from use for at least an hour prior to MRI to prevent nicotine withdrawal interference with MRI.

### 2.3 Measures

#### 2.3.1 Customary drinking and drug Use record

To determine lifetime patterns of drug and alcohol use, participants were given the Customary Drinking and Drug Use Record (CDDR) (65) at baseline to measure maximum frequency of substance use, substance abuse and dependence symptoms, and the age of onset for first and regular (defined as weekly for one year) use.

#### 2.3.2 Timeline follow-back

A modified version of the Timeline Follow-Back (TLFB) interviews were conducted to measure substance use patterns on a weekly basis for the past year while providing memory cues such as holidays and personal events (66, 67). Substances were measured by standard units [alcohol (standard drinks), nicotine (number of cigarettes and hits of chew/snuff/pipe/cigar/hookah), cannabis (smoked/vaped [f_l]ower, concentrates, edibles were measured and dosing was converted to joints based grams), ecstasy (number of tablets), sedatives (number of pills or hits of gamma-hydroxybutyrate), stimulants (cocaine and methamphetamine use converted to milligrams and number of amphetamine pills), hallucinogens (number of hits or occasions of ketamine/salvia/shrooms/other hallucinogens), opioids (number of hits of heroin/opium), and inhalants (number of hits)].

#### 2.3.3 Drug toxicology

Abstinence was evaluated at each session through urine toxicology. The ACCUTEST SplitCup 10 Panel drug test measures ten substances, including THC. All urine samples were also tested using NicAlert to test cotinine level, a metabolite of nicotine. Participants also wore PharmChek Drugs of Abuse Patches, which continuously monitor sweat toxicology for the presence of ten substances, including THC. Participants also underwent breathalyzer screens to test for alcohol use at the start of each session.

#### 2.3.4 Wide range achievement test-fourth edition

The Wide Range Achievement Test-Fourth Edition (WRAT-4) word reading subtest estimated quality of education and intelligence through word recognition (68, 69).

#### 2.3.5 fMRI affective Go/No-go task

Participants completed a Go/No-go task featuring faces expressing happy, fearful, or calmly emotions (i.e., positive, negative, and neutral), designed by the Sackler Institute for Developmental Psychobiology (23, 70). For this paradigm, two facial expressions were used within a trial. Using a rapid event-related design, participants were instructed which stimuli (i.e., expression) to respond to through pressing the target box (Go) and what stimuli they should withhold responding by not pressing the target box (No-go). For each trial, faces would appear for 500 milliseconds followed by jittered interstimulus interval from 2 to 14.5 s in duration. Participants were exposed to 48 total stimuli which were presented in a pseudorandomized order (35 “Go” stimuli and 13 “No-go” stimuli) in each trial. In one run, Participants completed a total of six trials which permitted every combination of positive, negative, and neutral expressions to serve as either Go or No-go stimuli for each participant. Directions for the task were to respond as fast as possible and to not wait for the stimuli to disappear, while also making as few errors as possible.

#### 2.3.6 MRI Pre-processing

See Supplementary Materials for MRI acquisition methodology; applicable for all subjects. Data was processed using Analysis of Functional NeuroImages [AFNI (71)] and Matlab (72). Images were processed through standard preprocessing pipelines within AFNI (i.e., “afni_proc.py”). The first three repetition times (TRs) were removed to eliminate initial scanner noise. To account for low and high frequency artefactual signals caused by head motion, physiological changes, and hardware instabilities, the time series per each voxel were “despiked” and these isolated spikes were replaced to fit the modeled data for the voxel using “3dDespike”. Voxel time series were corrected to align all acquired data to the same temporal spot of origin through “3dTshift”. To further limit head motion within BOLD signaling, volumes were registered based on the volume run with the least amount of motion artefacts within the dataset and then warped into standard Montreal Neurological Institute (MNI) coordinate space (73) through “3dVolreg”. Motion censoring was employed when 10% or more of head motion was observed as based on previous literature of similar samples (61). Data was spatially smoothed using a Gaussian function with the default 4 mm full width at half maximum (FWHM) with “3dmerge”. Voxels were scaled to a mean of 100 for interpretative purposes.

Functional responding to correct positive Go and No-go trials was examined against functional responding to correct neutral Go or No-go trials, respectively. These trials were first deconvolved with a gamma-variate hemodynamic response function (HRF) for each individual participant with AFNI’s 3dDeconvolve (74), while accounting for six motion parameters, incorrect trials, and trials of no-interest by regressing them out (75). In this way, analyses examined only functional activation for correct responding to the positive Go or No-go stimuli and not incorrect responding.

### 2.4 Statistical analysis

#### 2.4.1 Sociodemographic, substance use, and behavioral task performance

Sociodemographic and substance use variables were examined using ANOVAs and Chi-square tests in *R* (76). Behavioral task performance [i.e., % commission errors, % omission errors, response time (RT)] was analyzed to determine potential group differences in responding with a series of ANOVAs, then rerun covarying for past year alcohol use and cotinine levels. Decisions for statistical significance were determined at *p* = .05 for all sociodemographic, substance use, and behavioral analyses.

#### 2.4.2 fMRI BOLD group analysis

Whole-brain analyses examining BOLD responses to correct positive stimuli against correct positive stimuli were conducted at the group-level through a voxel-by-voxel ANCOVA with “3dttest++” for: (1) all face condition (i.e., both go and no-go contrast); (2) Go conditions; and, (3) No-go conditions, while controlling for past-year alcohol use and cotinine level on the day of fMRI scanning, across the whole sample. A family-wise error (FWE) threshold of *p*_FWE_<0.05 and an individual voxel threshold at *p* < 0.001 was applied to all models using a clusterthreshold method of correcting for multiple comparisons using Monte Carlo simulations within 3dClustSim (77), resulting in a cluster threshold of 18.0 at third-nearest-neighbor clustering. This methodology for cluster thresholding has been shown to effectively control false-positive rates (78, 79).

#### 2.4.3 gPPI analysis

A generalized Psychophysiological Interaction (gPPI) was conducted to examine context-specific functional connectivity between the ventral striatum and other brain regions between groups (80). gPPI methodology allows examination of the interactions amongst connectivity between participant variables (i.e., cannabis group) and physiological variables (i.e., HRF) (81). gPPI analyses were conducted with a bilateral anatomical ventral striatum seed, ascertained using automated anatomical labelling atlas (82). (1) Beta-values from clusters were first extracted and statistically examined across groups using a region-of-interest (ROI) approach which was conducted with general linear models, while controlling for past year alcohol and cotinine levels. (2) The gPPI connectivity analysis was completed by taking the average time series for the seed region, running deconvolution on the seed’s time series in the preprocessing stage and creating interaction regressors. Interaction regressors were re-convolved using the gamma HRF and concatenated across runs. AFNI’s “3dttest++” was rerun adding these two regressors to the models defined above. Statistical correction for multiple comparisons was consistent with the group analyses mentioned.

## 3 Results

### 3.1 Sociodemographic and substance Use data

There were no differences between cannabis-using and control groups in age (*p* = .53), gender distribution (*p* = .052), ethnicity (*p* = .31), race (*p* = .48), educational attainment (*p* = .33) and WRAT-4 Word Reading (*p* = .62). As expected, there were differences in lifetime [*F*(1,66) = 24.6, *p* < .001] and past-year cannabis use [*F*(1,66) = 30.4, *p* < .001], past-year tobacco use [*F* (1,66) = 5.4, *p* = .02], cotinine levels at MRI [*F*(1,66) = 4.0, *p* = .049], and alcohol consumed within the past-year [*F* (1,66) = 16.6, *p* < .001] (see Table 1); thus, past-year alcohol and cotinine levels were included as covariates in all fMRI analyses.

### 3.2 Behavioral data

Cannabis-using participants did not significantly differ on the number of commission errors, omission errors, Go RTs, and No-go RTs when compared to control participants (all *p*’s > .05) (see Table 2) and maintained after controlling for past-year alcohol use and cotinine level (all *p*’s > .05).

### 3.3 fMRI whole-brain BOLD response

#### 3.3.1 Overall positive > neutral face effects

Cannabis-using participants did not display any clusters of increased or decreased BOLD activation compared to controls when looking at response to all positive > neutral faces (i.e., regardless of condition).

#### 3.3.2 Positive go > neutral go effects

**FIGURE 1:**
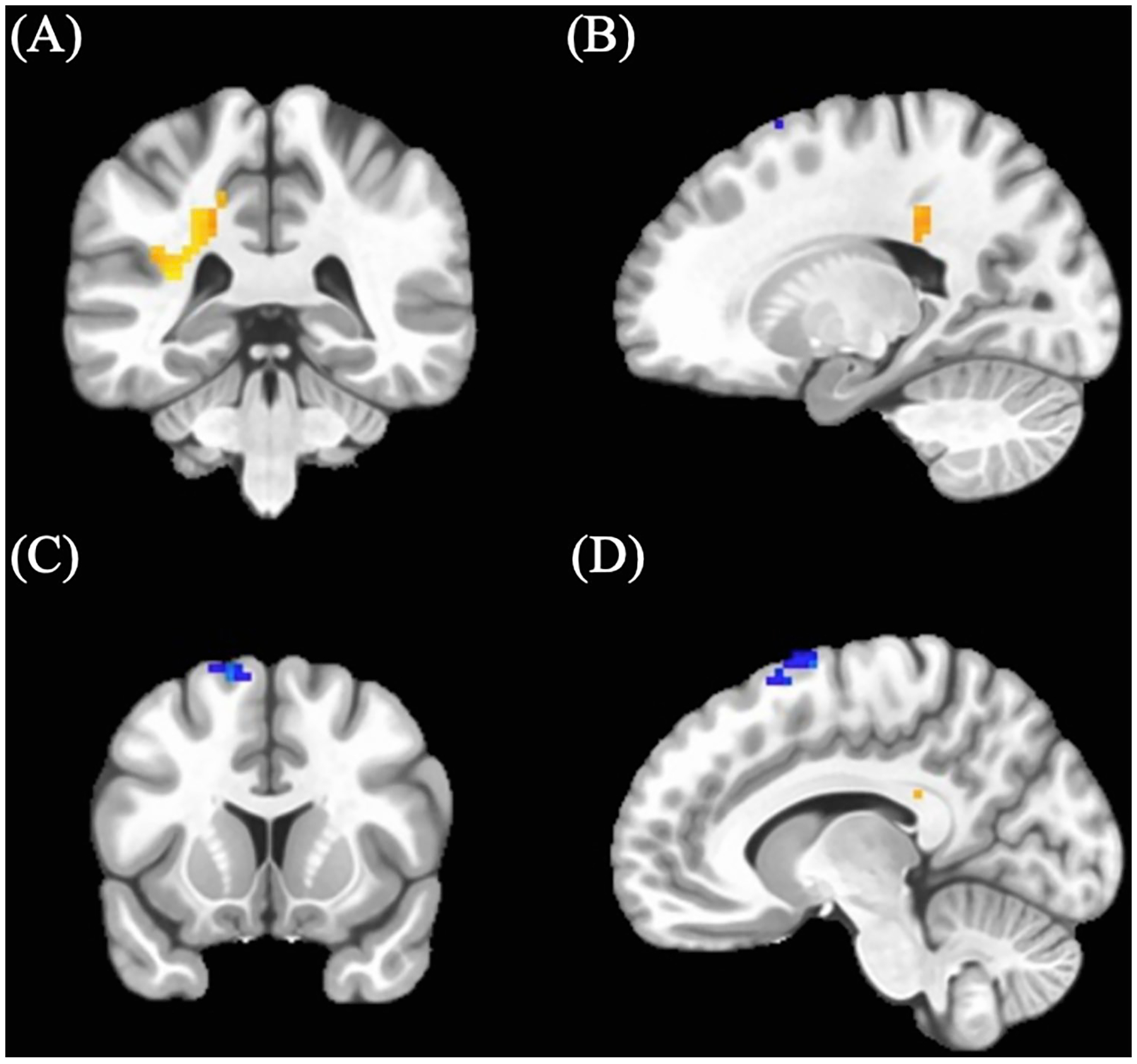
Significant positive n> neutral Go BOLD clusters between cannabis-using and non-using participants. (A) Coronal and (B) sagittal view of left middle cingulum cluster demonstrating increased activation amongst cannabis-using participants. (C) Coronal and (D) sagittal view of left supplemental motor cluster demonstrating decreased activation amongst cannabis-using participants.

Cannabis-using participants showed a cluster of increased BOLD response (M = 0.05, SD = 0.037) in an area including the left middle cingulum (see Table 3; Figure 1) relative to control participants (M = 0.02, SD = 0.068). In addition, cannabis-using participants displayed a cluster of decreased BOLD activation (M = 0.13, SD = 0.21) in the left supplemental motor area (see Table 3; Figure 1) relative to controls (controls: M = 0.19, SD = 0.31).

#### 3.3.3 Positive no-go > neutral no-go effects

**FIGURE 2:**
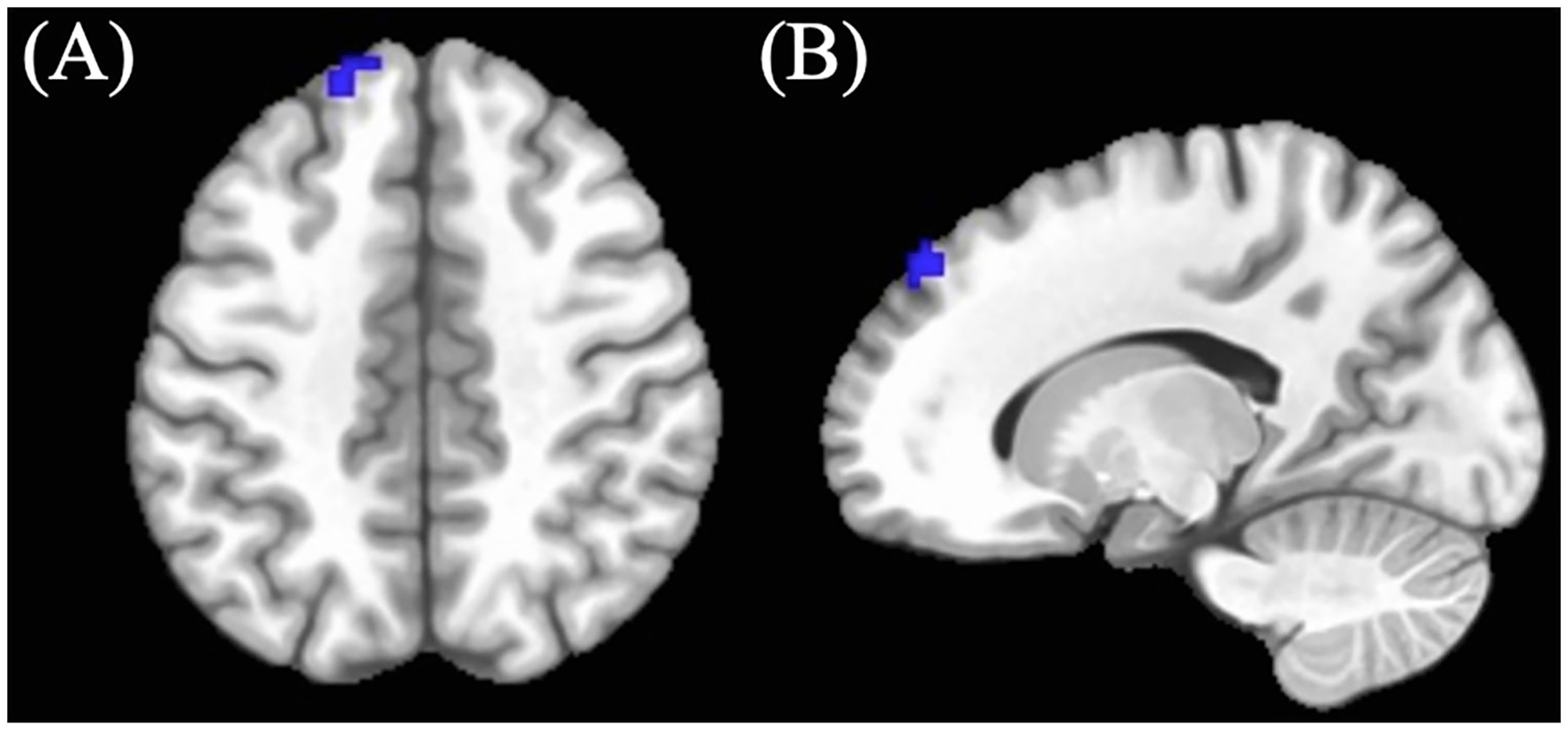
Significant positive > neutral No-go BOLD cluster between cannabis-using and non-using participants. (A) Axial and (B) sagittal view of left superior frontal cluster demonstrating decreased BOLD response in cannabis-using group relative to non-using group.

Cannabis-using participants showed decreased BOLD response (M = −0.25, SD = 0.43) in the left superior frontal region compared to controls in the positive > neutral no-go condition (see Table 3; Figure 2) (controls: M = −0.01, SD = 0.43). (See Table 3).

#### 3.3.4 Covariate effects

More past year alcohol use was associated with greater BOLD activity clusters in the right postcentral, and right and left middle cingulum regions in positive > neutral Go conditions. Higher cotinine level was associated with less BOLD activity in the left medial orbitofrontal region and greater BOLD activity in the right superior frontal region in positive > neutral Go conditions. (See Table 3).

### 3.4 Bilateral ventral striatal seed activation

#### 3.4.1 Overall positive > neutral faces

Cannabis-using participants did not differ from controls in ventral striatal activity during all positive face conditions (*p* = .25).

#### 3.4.2 Positive > neutral go

**FIGURE 3:**
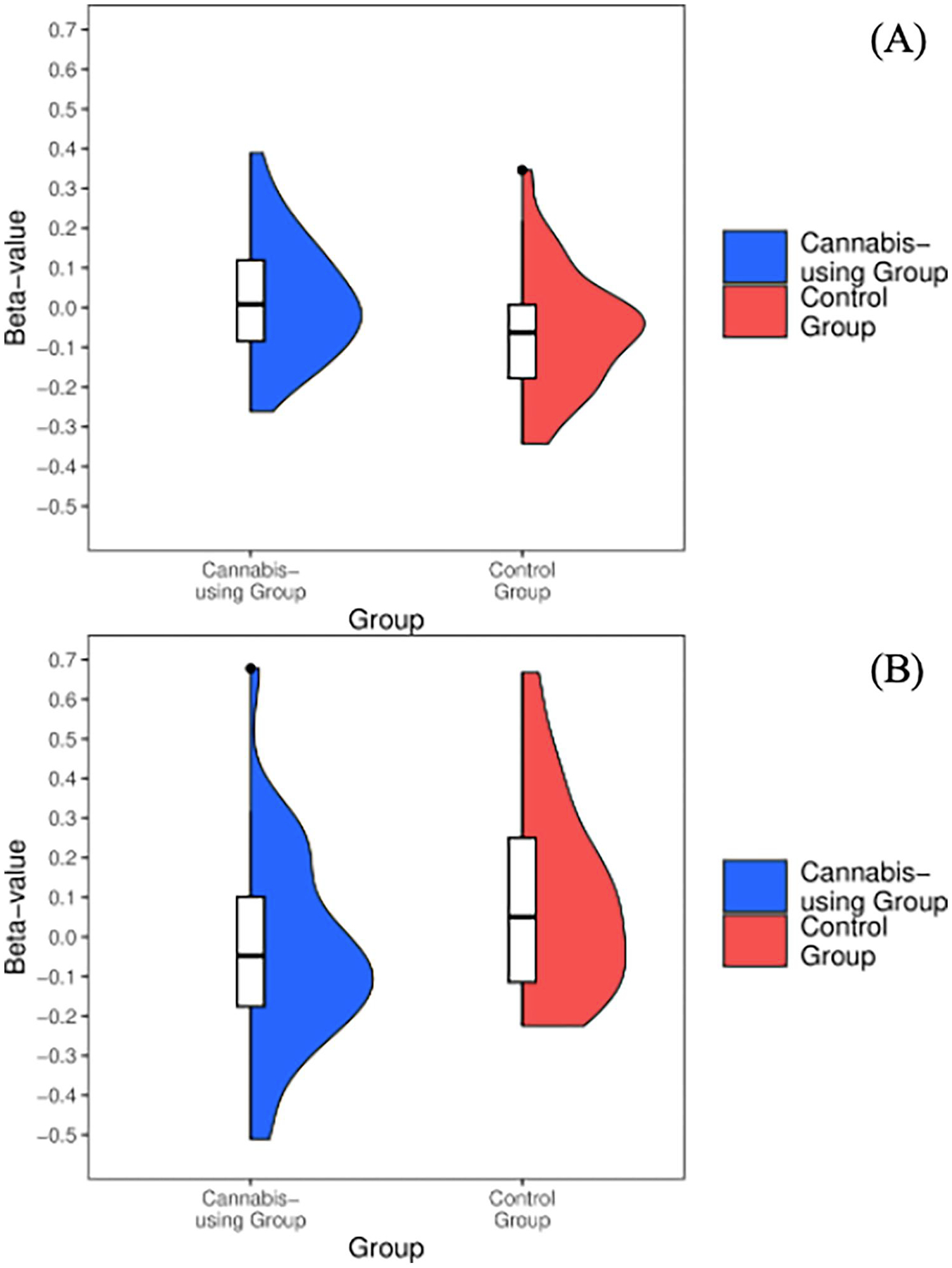
Significant ventral striatum activity for (A) positive > neutral Go and (B) positive > neutral No-go conditions for cannabis-using and non-using groups.

Cannabis-using participants demonstrated increased (M = 0.02, SD = 0.15) activation in the ventral striatum during positive Go conditions [*t*(64)=−2.24, *p* = 0.02] when compared to controls (M = −0.06,SD = 0.15) (see Figure 3A).

#### 3.4.3 Positive > neutral No-Go

Cannabis-using participants demonstrated decreased activation (M = −0.04, SD = 0.24) in bilateral ventral striatum during positive No-go conditions [*t*(64) = 2.62, *p* = 0.01] when compared to controls (M = 0.1, SD = 0.24) (see Figure 3B). No effects of covariates were observed.

### 3.5 Bilateral ventral striatal seed connectivity (gPPI)

Across all conditions (i.e., overall positive > neutral faces, positive > neutral Go, and positive > neutral No-go) no clusters of connectivity with the bilateral ventral striatum seed survived thresholding.

## 5 Discussion

Cannabis is one of the more commonly used substances in the United States (1, 2), thus, understanding the impact of cannabis use on neurodevelopment amongst adolescents and young adults is of increasing importance. Prior research has shown aberrant associations between cannabis use and reward circuitry activity (30, 40, 41, 44, 45); yet minimal studies have investigated response to positive stimuli during inhibitory control conditions in abstinent cannabis-using adolescents and young adults. In the present study, we found that cannabis-using participants demonstrated increased BOLD response in left middle cingulum and decreased BOLD response in left supplementary motor area while engaging in positive viewing (i.e., happy Go), despite similar behavioral performance. In positive inhibition (i.e., happy No-go), cannabis-using participants displayed decreased activation in a cluster within the left superior frontal region, relative to controls. In addition, an ROI activation analysis revealed abstinent cannabis-using participants demonstrated hyperactivity during engagement of positive stimuli and hypoactivity during inhibition of positive stimuli within the ventral striatum compared to controls; although, when conducting a context-dependent connectivity analysis, no connectivity differences were observed.

Cannabis-using adolescents and young adults displayed increased activation in the left middle cingulum and ventral striatum, and decreased activation in left supplemental motor area relative to control participants while freely responding to positive faces (i.e., happy Go). The cluster identified as the left middle cingulum is positioned primarily within anterior cingulum white matter tract, which corresponds with increased cingulate activity when viewing positive faces (52), though, also shares proximity with the inferior parietal region. Notably, the inferior parietal region has been implicated in cannabis fMRI investigations, demonstrating increased BOLD responding in Go conditions among adolescents (61), decreased BOLD responding in positive word conditions in young adults (83), and aberrant inferior parietal-cerebellar connectivity on a Go/No-go task (84, 85). As this cluster additionally sits within the anterior cingulum bundle, it is worth discussing the relevance of this primarily white-matter activity finding as well given that white matter exhibits activation and functional BOLD differences are worthy of documentation (i.e., instead of the approach to censor for solely grey matter activation) (86). It is possible that activation within these white matter may represent broader recruitment of cingulum and surrounding regions for task performance, and their corresponding communicating fibers, however, this should be further investigated using network-based analyses of task-related BOLD data to comprehensively examine gray and white matter contributions to these group differences. Notwithstanding, research has identified impacted cingulum development due to cannabis use across young adults (87) with reduced structural volumes observed (88, 89). Notably, the anterior cingulum plays a role in both executive functioning and emotion processing (90) and has been implicated in Go/No-go task performance within community samples (91). Follow-up analyses should continue to investigation structural/functional network-related characteristics of cannabis use disorder in adolescence.

Analyses additionally observed increased ventral striatum activity during positive response conditions, which corresponds with hyperactivation seen during the reward outcome trials on the MID task among cannabis-using groups (30, 45, 46). This may possibly represent shared activation patterns for positive facial stimuli and monetary reward stimuli. Hyperactive regions observed within our cannabis group may be indicative of increased recruitment in order to complete the task similar to their non-using peers (i.e., maintain rule set to initiate motor response to positive faces); alternatively, increased activation in cingulum and ventral striatum regions could demonstrate sensitized responses to reward processing within cannabis-using groups, despite similar behavioral performance and abstinence length. Lastly, decreased left supplemental motor activation observed is consistent with findings specifically on tasks requiring motor response (92, 93). As all three regions (left middle cingulum, left supplemental motor, and ventral striatum) are rich in CB1 receptors (94, 95), aberrant activation in these areas may be suggestive of compensatory mechanisms by which cannabis-using participants are more heavily recruiting middle cingulum and ventral striatal regions due to positive nature of task condition, while non-using controls more readily engage supplementary motor areas to respond to positive stimuli.

Decreased left superior frontal and ventral striatum activations were observed in positive response inhibition (i.e., happy No-go) for cannabis-using participants relative to controls. Studies of BOLD activation elicited by inhibitory tasks have shown that cannabis-using groups generally display aberrant BOLD response in frontal regions (59, 61, 84, 96). In a similar sample, Wallace et al. (13) found increased activation in left frontal gyrus when examining response inhibition to calm (i.e., neutral) faces. Further, given the mixed findings on BOLD response elicited by positive stimuli (52, 56, 57), we demonstrate a unique pattern of left superior frontal activation in inhibitory trials with added component of inhibition *to* positive stimuli. While we do see in non-using teens that more frontal activation is recruited for successful inhibitory response to positive faces (21, 23), it is hypothesized that cannabis-using participants may not be actively recruiting frontal regions; further evidenced by connectivity studies of reward paradigms, where frontal connectivity network differences are observed even following a period of monitored abstinence (40, 41). Hypoactivation was observed in positive inhibition trials which additionally aligns with previous MID studies of reward anticipation (30, 44–46), however, as this task did not involve “anticipation” *per se*, these findings may instead represent discrepant BOLD responses elicited by positive inhibition. Findings could be attributed to more effortful inhibition of the ventral striatum when cannabis-using individuals are asked to inhibit response to positive (i.e., rewarding) faces in order to complete the task (relative to the non-using literature 97). Overall, the positive inhibition findings may represent that when cognitive control components are added it could disrupt typical reward processing. Future studies with tasks engaging both inhibition and reward processing are needed as these could present more ecologically valid day-to-day interactions with rewarding stimuli.

Interestingly, we did not observe connectivity differences with bilateral ventral striatal activity and other cortical regions between cannabis-using and non-using participants, despite observing group differences on ventral striatal activity—which was dependent on task condition. Null findings align with ventral striatal connectivity differences that are largely recovered after one month of cannabis abstinence in young adult males (42). Indeed, our own sample—on average—had 31 days of abstinence at the time of MRI. Thus, it is possible that frontostriatal connectivity differences are resolved after at least two-weeks of monitored abstinence, and differences would be more evident with shorter durations of abstinence (15, 40, 41). Yet, the present analyses did observe varying ventral striatal activity dependent on task condition as described above. Correspondingly, research on the ventral striatum shows differential activation dependent on emotional valence (greater deactivation for inhibition to neutral compared to positive lexical stimuli) (97) and hyperactivation for positive faces in Go trials compared to No-go trials in teens (23). These findings lend evidence to a larger theory of dysregulated ventral striatum activity within cannabis-using samples across fMRI tasks (30), positing the ventral striatum in either the risk for or consequence of cannabis use in this age range (98, 99).

Taken together, we observed discrepant BOLD response elicited by positive face responding in cannabis-using participants compared to controls both in various cortical regions necessary for task completion and specifically in reward-related structures implicated in positive task conditions (i.e., ventral striatum) (21, 23). These findings are evident following a monitored period of abstinence and thus may represent a consequence of prolonged cannabis use during these developmental periods and could ameliorate after longer sustained abstinence (12). More broadly, aberrant functional activation seen in cannabis-using individuals relative to controls across fMRI tasks has been implicated in the literature previously, particularly in tasks requiring increased attention, inhibition, and/or set shifting (11, 100, 101). This further posits the aforementioned mechanism of *compensation*—through dysregulation of CB1 receptors (12, 61, 102, 103)—to exhibit similar behavioral performance relative to their non-using peers, particularly in these high-density CB1 regions (94, 95). Specifically, significant regions in the present analysis are implicated in affective processing paradigms (21, 23, 90–92) and facilitate other task completion processes (e.g., motor component in Go trials), possibly lending evidence towards compensation. Additionally, a novel and interesting interplay between reward processing and inhibitory control is achieved with this task and more studies are needed to ascertain the contributions of each process in this relationship. Importantly, the culmination of these findings could have important implications for downstream impacts to treatment outcomes or interpersonal functioning, specifically in this neurodevelopmental period.

Notably, we did also observe unique covariate findings of interest. Past year alcohol use was associated with increased BOLD activation in right postcentral and bilateral cingulum regions in positive responding, relative to neutral responding, conditions. Most research on BOLD response to rewarding cues in alcohol-dependent individuals relates to alcohol-related cues; the present findings add-on to the literature demonstrating aberrant frontal activation as it relates to positive cues (104–106). Additionally, higher levels of cotinine at the time of scan were associated with BOLD activation decreases in left medial orbitofrontal and increases in right superior frontal regions. Minimal research has been conducted on nicotine in positive face responding, but reward-related findings have implicated blunted striatal reward (107) and generally altered amygdala function (108). Thus, these frontal cotinine findings observed demonstrate continued aberrant findings related to nicotine levels in frontolimbic regions. These provide rationale for the need of cannabis analyses to control for patterns related to polysubstance use. Further, these outcomes bolster general findings on aberrant brain activity in affective contexts seen across substance use more broadly (109, 110).

It is worth noting limitations of the present analysis. Notably, causality cannot be determined from the sample due to cannabis use initiation occurring prior to the study protocols. Longitudinal studies can assess functional activation elicited by affective processing tasks in substance-naïve youth and determine whether this represents a risk factor for cannabis use or is affected by use. Further, these studies can also investigate developmentally-dependent (i.e., age-related) changes in functional activation across adolescence to young adulthood in a larger longitudinal sample. Second, the sample was balanced for aerobic fitness and excluded individuals who could not undergo acute aerobic fitness measurements; this decreases generalizability to adolescent and young adult populations who may be primarily sedentary. Third, the study excluded for those with mood-related comorbidities, which allowed for more conclusive implications on cannabis use’s association with positive responding and inhibition but may be more varied in those with comorbid depressive disorders (111, 112). In line with the above, a sample willing to abstain from cannabis was recruited in order to examine cannabis effects that remain after withdrawal, this limits generalizability to individuals who may be unwilling or unable to sustain abstinence, such as those with more severe cannabis use disorder. Fifth, it is possible that gPPI connectivity analyses were underpowered (113) to detect small effect sizes and might explain null connectivity differences between cannabis-using and non-using participants. Finally, cannabis metabolites cycle out within a three-to-four week period (114); thus, future studies are needed to determine whether differences exist at the acute stage (i.e., no abstinence period) and if subtle differences would recover with longer periods of sustained abstinence.

The current study found that after at least two-weeks of monitored abstinence, cannabis-using adolescents and young adults displayed increased BOLD response in left middle cingulum and ventral striatum, and decreased BOLD response in left supplemental motor areas in positive Go conditions. In addition, decreased BOLD response in a left superior frontal cluster and ventral striatum in positive No-go conditions was observed in cannabis-using participants. These aberrant BOLD activations align with previous findings and may further implicate compensatory theories within cannabis-using individuals who are recruiting other functional regions to assist in responding to positive stimuli on a Go/No-go task or, may represent increased activation when responding to positive cues and disrupted functional response when an inhibitory control component is added. Ventral striatum outcomes correspond with monetary-incentive investigations (30), while also representing a novel result in cannabis research relative to the neurodevelopmental adolescent literature on positive affective processing (23). Further, we did not observe significant context-dependent connectivity differences between the ventral striatum and other cortical regions (15). Overall, these findings, coupled with the existing literature, suggest that BOLD activity elicited by either engagement or inhibition to positive faces—even following at least two-weeks of monitored abstinence—differs between cannabis-using and non-using adolescents and young adults. Jointly investigating response inhibition and reward processing in cannabis-using individuals may present more ecologically valid methods of examining contributions to escalation or continuation of use into adulthood, as this interplay is described within withdrawal stages of addiction models (33, 47). Future prospective, longitudinal studies, are needed to further elucidate the causal relationship between escalating cannabis use and functional activation elicited by positive stimuli, specifically examining the contributions of the ventral striatum in this relationship.

## Supplementary Material

supplementary table 1

The Supplementary Material for this article can be found online at: https://www.frontiersin.org/articles/10.3389/fradm.2026.1737529/full#supplementary-material

## Figures and Tables

**TABLE 1:** Sociodemographic, substance use, and behavioral characteristics.

*N*	Cannabis-using group	Control group
	35	33
***M* (SD), *N*, or %**		
Age	21.5 (2.1)	21.1 (2.6)
Sex (Female/Male)	13/22	21/12
Race (% Caucasian)	60.0%	69.7%
Ethnicity (% Non-Hisp)	77.1%	84.9%
Educational Attainment	14.0 (1.5)	14.5 (2.2)
WRAT-4 Word Reading	104.7 (13.0)	106.1 (11.0)
Past year Alcohol Use, standard drinks^a^*	325.0 (301.3)	87.7 (150.3)
Past year Tobacco Use, number of cigarettes/hits^a^*	189.8 (466.6)	0.5 (2.2)
Cotinine Level^b^*	1.9 (1.9)	1.2 (0.7)
Past year Cannabis Use, grams^a^*	429.7 (447.5)	–^d^
Lifetime Cannabis Use, uses^c^*	1,200.7 (1,389.0)	1.4 (4.0)
Age at Regular Cannabis Use Onset	17.5 (1.7)	–^d^
Cannabis Abstinence Length in days^e^	31.3 (23.2)	–^d^
Total Cannabis Use Abuse/Dependence Symptoms^f^	4.6 (1.8)	–^d^
Diagnosis of Cannabis Abuse or Dependence^f^	82.9%	–^d^

WRAT-4 – Wide Range Achievement Test-Fourth Edition.

a Measured in standard uses on TLFB (67).

b Measured at MRI Scan.

c Measured in standard uses on CDDR (65).

d Not applicable.

e Calculated from TLFB last CAN use date and date of fMRI.

f Determined through DSM-IV-TR criteria.

* *p* < .05.

**TABLE 2:** Behavioral task performance.

Performance metric	Cannabis-using group	Control group
Omission Errors to Positive Go Faces (%, ±S.D.)	0.8% ± 1.4	1.1% ± 1.8
Omission Errors to Neutral Go Faces (%, ±S.D.)	2.7% ± 8.5	4.9% ± 12.8
RT to Correct Positive Faces (ms, ±S.D.)	547.0 ± 91.6	528.0 ± 76.5
RT to Correct Neutral Faces (ms, ±S.D.)	574.0 ± 93.0	564.0 ± 103.0
Commission Errors to Positive No-go Faces (%, ±S.D.)	7.5% ± 7.5	8.9% ± 7.2
Commission Errors to Neutral No-go Faces (%, ±S.D.)	8.2% ± 8.03	12.0% ± 11.5
RT to Incorrect Positive No-go Faces (ms, ±S.D.)	399.0 ± 24.5	402.0 ± 36.6
RT to Incorrect Neutral No-go Faces (ms, ±S.D.)	409.0 ± 32.5	418.0 ± 32.5

RT, response time.

**TABLE 3:** Significant BOLD clusters.

Cluster #	Voxels	MNI coordinates^a^			Annotations^b^	Directionality
		Peak x	Peak y	Peak z		
**Cannabis Findings – Positive > Neutral Go**						
1	49	−22.5	−37.5	+34.5	Left Middle Cingulum	Cannabis-using > Control
2	26	−10.5	+7.5	+73.5	Left Supplemental Motor	Cannabis-using < Control
**Cannabis Findings – Positive > Neutral No-Go**						
1	18	−16.5	+49.5	+46.5	Left Superior Frontal	Cannabis-using < Control
**Alcohol Findings – Positive > Neutral Go**						
1	23	+64.5	−13.5	+34.5	Right Postcentral	↑ Alcohol associated with t activation
2	20	+ 19.5	−37.5	+34.5	Right Middle Cingulum	↑ Alcohol associated with f activation
3	18	−19.5	−37.5	+34.5	Left Middle Cingulum	↑ Alcohol associated with f activation
**Cotinine Findings – Positive > Neutral Go**						
1	23	−7.5	+22.5	−19.5	Left Medial Orbitofrontal	↑ Cotinine associated with f activation
2	19	+ 19.5	+25.5	+58.5	Right Superior Frontal	↑ Cotinine associated with i activation

a LPI coordinate order.

b Annotated using Automatic Atlas Labeling using “*label4MRI”* in R (82).

## Data Availability

The raw data supporting the conclusions of this article will be made available by the authors, without undue reservation.
