# Supplementary material for: Positive face BOLD response and task-dependent ventral striatal functional connectivity during Go/No-go task among abstinent cannabis-using adolescents and young adults
Source: Front Adolesc Med. Author manuscript; Available in PMC 2026 Mar 11. (PMC12975031; doi:10.3389/fradm.2026.1737529)
Supplement: supplementary table 1 [file NIHMS2152530-supplement-supplementary_table_1.docx]

Supplementary Material

# Supplementary Methods

*MRI acquisition—*MRI scans were acquired on a 3T Signa LX MRI scanner (GE Healthcare, Waukesha, WI) using a 32-channel quadrature transmit/receive head coil. High-resolution anatomical images were acquired using a T1-weighted spoiled gradient-recalled at steady-state (SPGR) pulse sequence (TR=8.2 ms, TE=3.4 s, TI=450 and flip angle of 12°). The in-plane resolution of the anatomical images was 256x256 with a square field of view (FOV) of 240 mm. One hundred fifty slices were acquired at 1 mm thickness. Echoplanar images (EPI) were collected while performing the Affective Go/No-go task [designed using E-Prime software ^110^, see below] using a T2*-weighted gradient-echo EPI pulse sequence (TR/TE=2500ms/30ms, FOV=200cm, matrix 64x64 pixels, slice-thickness=3.2mm, flip angle=90 degrees, 44 contiguous sag slices).
